# Should Artificial Intelligence be used to support clinical ethical decision-making? A systematic review of reasons

**DOI:** 10.1186/s12910-023-00929-6

**Published:** 2023-07-06

**Authors:** Lasse Benzinger, Frank Ursin, Wolf-Tilo Balke, Tim Kacprowski, Sabine Salloch

**Affiliations:** 1 Institute for Ethics, History and Philosophy of Medicine, Hannover Medical School (MHH), Carl-Neuberg-Str. 1, 30625 Hannover, Germany; 2 Institute for Information Systems, TU Braunschweig, Braunschweig, Germany; 3 Division Data Science in Biomedicine, Peter L. Reichertz Institute for Medical Informatics of Technische Universität Braunschweig and Hannover Medical School, Braunschweig, Germany; 4 Braunschweig Integrated Centre for Systems Biology (BRICS), TU Braunschweig, Braunschweig, Germany

**Keywords:** Ethics, Clinical, Decision-making, artificial intelligence, Decision support systems, clinical, Systematic review

## Abstract

**Background:**

Healthcare providers have to make ethically complex clinical decisions which may be a source of stress. Researchers have recently introduced Artificial Intelligence (AI)-based applications to assist in clinical ethical decision-making. However, the use of such tools is controversial. This review aims to provide a comprehensive overview of the reasons given in the academic literature for and against their use.

**Methods:**

PubMed, Web of Science, Philpapers.org and Google Scholar were searched for all relevant publications. The resulting set of publications was title and abstract screened according to defined inclusion and exclusion criteria, resulting in 44 papers whose full texts were analysed using the Kuckartz method of qualitative text analysis.

**Results:**

Artificial Intelligence might increase patient autonomy by improving the accuracy of predictions and allowing patients to receive their preferred treatment. It is thought to increase beneficence by providing reliable information, thereby, supporting surrogate decision-making. Some authors fear that reducing ethical decision-making to statistical correlations may limit autonomy. Others argue that AI may not be able to replicate the process of ethical deliberation because it lacks human characteristics. Concerns have been raised about issues of justice, as AI may replicate existing biases in the decision-making process.

**Conclusions:**

The prospective benefits of using AI in clinical ethical decision-making are manifold, but its development and use should be undertaken carefully to avoid ethical pitfalls. Several issues that are central to the discussion of Clinical Decision Support Systems, such as justice, explicability or human–machine interaction, have been neglected in the debate on AI for clinical ethics so far.

**Trial registration:**

This review is registered at Open Science Framework (https://osf.io/wvcs9).

**Supplementary Information:**

The online version contains supplementary material available at 10.1186/s12910-023-00929-6.

## Background

Being a physician means making decisions. Many of the clinical decisions in daily practice are value-laden, and some of them carry a clear ethical component, for example, when healthcare professionals need to decide for incapacitated patients or in potentially controversial settings, such as abortion or transplantation medicine. According to empirical studies, physicians rather frequently encounter ethical challenges that often relate to impaired decision-making capacity, disagreement with the patients’ family members or decision-making at the end of life [1–3].

Both physicians and nurses report increased levels of stress in cases in which they feel unfit to oversee moral decision-making. This may occur due to insufficient ethical education or restrictive institutional standards [4]. The stress perceived can lead to burnout in healthcare personnel, and even to their resignation in extreme cases [5]. Moral distress, thus, can negatively affect the quality of healthcare and the lives of healthcare providers.

Clinical Ethics Support Services (CESS) are long-standing, established structures providing support and training for the adequate dealing with moral challenges in clinical practice. Clinical ethicists can, if required, support physicians and other healthcare personnel, as well as patients, their families or other stakeholders, with ethical expertise in challenging instances of, for example, end-of-life decision-making or futility of treatment. Ethical services in the clinic have proven their value over time [6, 7]. The coverage of ethics services varies internationally [3, 8, 9]. Furthermore, the quality of CESS, and how to validate it, is disputed [10].

When it comes to medical decisions, the use of Artificial Intelligence (AI) is no longer a novelty in clinical practice. Diagnostics in various fields have been improved through the means of AI. Machine learning (ML)-powered tools are able to compete with or even outperform professionals in medical specialities such as radiology [11], cardiology [12] or dermatology [13]. Additional development is being made on the back of ML to improve diagnostics, monitoring and decision-making further. Moreover, clinical decision support systems (CDSS) have been a driving force in improving the precision of healthcare for patients by ensuring that as much available data as possible is considered in clinical decision-making [14]. In this sense, computer-based systems have shown the potential to skyrocket the availability and quality of individualised medicine.

The idea of getting computers to make morally acceptable decisions is not new either – at least to the community of ethicists. The term “moral robots” describes computer systems that operate in conformity with an ethical framework that they have been “taught” and, thereby, evaluate all their decisions for infringements of the said framework before executing them [15]. These decisions may have an impact on, for example, financial matters, issues of public security and the care of elderly people through robotic assistants.

A merging of the three different discourses on clinical ethics support, CDSS and artificial moral agents can be observed currently in the introduction of systems that use advanced data science for the support of ethical decision-making in clinical practice. Anderson and Anderson introduced MedEthEx, a medical ethical advisor based on conventional computer algorithms, in 2006, but never passed the testing stage [16]. More recently, conceptualized applications, such as Meier et al.’s METHAD, are being developed to prove their feasibility [17]. Envisioned uses of other hypothetical systems include support in difficult cases, everyday cases where ethics consultation is not available and in medical ethical education. Thus, although this idea may not be exactly new, the rapid development in computer sciences and the resulting new possibilities for data collection and processing enable promising progress in making the concept become a reality.

However, with new technology, new questions arise: what are the implications of supporting ethical decision-support with AI? Does the use of AI provide benefits in the clinical routine? How does it influence different stakeholders? And, as a basic requirement: should these tools be used at all? A systematic overview of the reasons for and against the use of ML for ethical decision support is missing so far, but is important to accompany the emergent debate early on. This systematic review, to the best of our knowledge, is the first to provide an overview of the ethical debate concerning reasons for and against the use of ML to support clinical ethical decision-making. It offers a summary of all the reasons given in the academic literature to date and might serve as a baseline study for future research and development.

## Methods

This systematic review of reasons provides a full cross-sectional profile of the current state of the ethical debate regarding the use of ML tools in clinical ethical decision-making. It condenses all the ethical reasons that have been given in academic journals regarding the topic at hand. It was conducted and reported conforming to the PRISMA-Ethics Reporting Guideline (see file “Additional File PRISMA-Ethics Reporting Guideline”) [18]. The review was pre-registered with Open Science Framework.

The search was carried out in four databases: PubMed, Web of Science, Google Scholar and Philpapers.org. The first three were included because they are the most comprehensive databases available, in order to provide a full overview of the debate. Philpapers.org was used with the main intention of including the philosophical side of the scientific debate. Additionally, LIVIVO was searched for potential book sources. However, it turned out that no relevant book sources could be identified, possibly due to the rather cutting-edge nature of the topic at hand. LIVIVO was, therefore, excluded from this review.

All database searches were conducted in September 2022. The search strings represent two semantic clusters: the technical cluster, which includes terms for the technological basis of the applications used, and the ethical cluster, which limits the purpose of the applications used to a clinical-ethical nature. The search strings are shown in Table 1.

**Table 1:** Search strings

PubMed	“((Artificial Intelligence) OR (Machine Learning) OR (clinical decision support) OR (AI advisor) OR (AI-driven decision support system) OR (Machine Learning-based clinical decision support)) AND ((algorithms[MeSH Terms]) OR (Artificial Intelligence[MeSH Terms])) AND ((ethics, clinical) OR (digital bioethics) OR (computational bioethics) OR (morals) OR (values)) AND ((ethics, clinical*[MeSH Terms]) OR (ethics, medical[MeSH Terms]) OR (ethics[MeSH Terms])) OR (Ethics, Professional[MeSH Terms]) OR (Decision Making/ethics[MeSH Terms])”
Web of Science	“ALL = (decision making) AND (ALL = (Machine Learning) OR ALL = (Artificial Intelligence) OR ALL = (algorithms) OR ALL = (Automated decision support) OR ALL = (AI advisors)) AND WC = (Ethics)”
Google Scholar	“("clinical ethics" | "digital bioethics") AND ("Artificial Intelligence" | "Machine Learning" | "clinical decision support") AND "clinical decision making"”
Philpapers.org	"(ethics|"digital_bioethics"|” computational_bioethics") + ("Machine_Learning"|”clinical_decision_support" | "AI_advisor" | "AI-driven_decision_support_system" | "Machine_Learning-based_clinical_decision_support")"

Inclusion and exclusion criteria were defined in advance (see Table 2). Articles were only included if they explicitly discussed the use of tools based on AI or ML. We considered a decision to be ‘clinical’ if it has consequences for individual patients at the bedside, and ‘ethical’ if it is concerned primarily with making decisions in an ethically (more) correct way. The medical discipline in which the decisions were made was not relevant, nor was the time period in which the literature was published. One exclusion criterion related to publications on the use of AI/ML in healthcare without an explicit intention to address its ethical dimension.

**Table 2:** Inclusion and exclusion criteria

Inclusion criteria	Exclusion criteria
• Dealing with AI/ML • Dealing with clinical ethical decision-making • Article published in a peer-reviewed scientific journal • English or German	• Publication on the use of AI/ML for not inherently ethical tasks (e.g. imaging diagnostics)

**Fig. 1:**
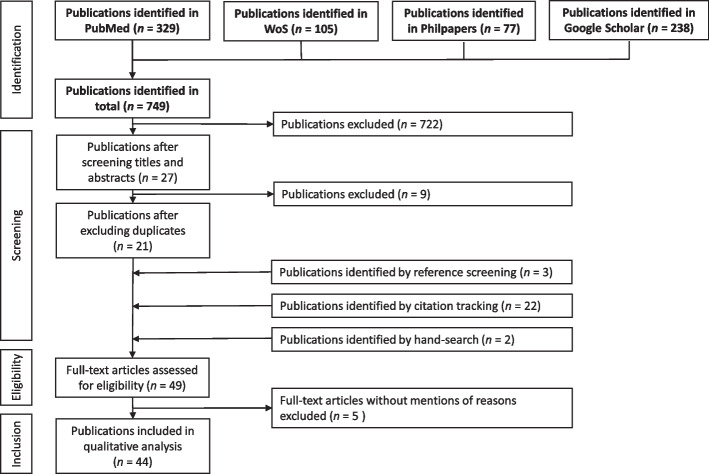
Flowchart of the literature selection

The publications retrieved in this way were then checked for duplicates before their references were searched for further relevant publications. LB reviewed the titles and abstracts of all articles identified against the criteria defined. Ambiguous cases were discussed with SS and FU to reach consensus. Given that the review addresses a topic at a relatively early stage of its development, we also checked whether the publications included so far had been cited in more recent articles, in order to find any recent publications that might not yet have been properly categorised. In addition, relevant publications found through searching by hand were included. Figure 1 shows a flowchart of the entire process.

The full-text screening and analysis was carried out by LB. Articles were searched for arguments for and against the use of ML tools in clinical ethical decision-making. If none were found, the article was excluded. Ambiguous cases were discussed again by the research team until consensus was reached.

The analysis of the full texts was conducted based on the methodology of the qualitative text analysis according to Kuckartz [19], using MAXQDA Plus 18. In accordance with Kuckartz’ mixed deductive and inductive approach, the authors first developed six main categories deductively (concept-driven). They consist of the four well-established principles of biomedical ethics by Beauchamp and Childress (autonomy, beneficence, non-maleficence, justice) [20], the additional principle of “explicability” that further captured the ethics of AI [21], and the category “other” for reasons that did not fit any of the categories mentioned. During the subsequent full-text analysis, these broad categories were further explicated inductively (data-driven) with complementary narrow categories that were applied to each individual reason in the documents analysed. Confining the narrow categories to be as specific and nuanced as reasonably possible allowed for a more small-sectioned review and analysis of the material. SS and FU revised any unclear cases.

Since quality appraisals are notoriously hard to come by in the field of ethics, we decided not to conduct one. Whether an assessment of the quality of ethical reasons is possible at all is the subject of ongoing scientific debates, but, so far, no standard has been universally agreed on [22].

## Results

**Fig. 2:**
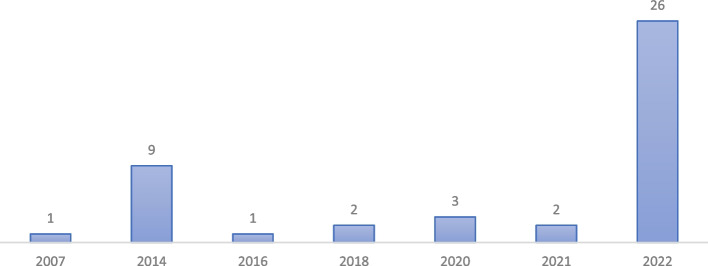
Number of publications included per year

Forty-four publications were analysed in this systematic review, the full listing can be found in “Additional File Included Publications”. The sample consists predominantly of journal articles (*n* = 24) or commentaries (*n* = 20), mostly published in the American Journal of Bioethics (*n* = 16), the Journal of Medical Ethics (*n* = 10) and the Journal of Medicine and Philosophy (*n* = 9). One of the articles was based on empirical research. The first authors of the publications analysed were mainly affiliated with institutions in the United States (*n* = 28) and Europe (*n* = 14), with only two affiliations being in South Africa (*n* = 1) and South Korea (*n* = 1). Figure 2 shows the profile of the years in which the articles were published, and confirms the rather recent emergence of the topic. The clusters around the years 2014 and 2022 are related to the first articles on the Patient Preference Predictor and METHAD, to which the majority of the publications included refer. These and the other AI tools discussed in the sample are characterised in Table 3.

**Table 3:** AI applications for clinical ethical decision-making as occurring in the sample

Name	Technological Basis	Field of Application	Use	Implemented/Conjectured
Medical Ethical Advisor (METHAD) [17]	Machine Learning, Fuzzy Cognitive Maps	General clinical practice, education	- encompasses the bioethical principles (Beauchamp, Childress) [20] in machine-readable form - input: patient status and preferences in machine-comparable values - general evaluation; numerical value of zero (against) to one (in favour of)	Implemented (“proof of concept”)
Patient Preference Predictor (PPP) [23]	Machine Learning, Population-based	General clinical practice, incapacitated patients	- takes defining characteristics and circumstances of the patient in question and empirical data on treatment preferences into account - approximates preferences of incapacitated patients regarding treatments	Conjectured
Do not attempt resuscitation—Algorithm (DNAR) [24]	Machine Learning	Emergency medicine	- predicts patients’ preferences on resuscitation measures in emergency situations - compares the patient’s data with that of other patients	Conjectured
Surgery Algorithm [25]	Machine Learning	Surgery	- strives to de-bias decision-making in the selection of patients for major surgery - gives an objective and equitable risk assessment for the patients - improves i.a. racial and socioeconomic justice	Conjectured
Autonomy Algorithm [26]	Machine Learning, based on healthcare records and social media	General clinical practice, incapacitated patients	- harvests information on patients with impaired capacity - predicts their preferences on important healthcare decisions	Conjectured

Hereafter, the reasons extracted will be presented in relation to the ethical principles with which they are associated. The focus is on the most prominent reasons that played the biggest role in the ethical debate on AI in clinical ethical decision-making. All reasons identified and their frequency of occurrence are specified in the file “Additional File List of Codes”.

A major result from the analysis is the fact that the scientific community has big hopes for AI regarding the enhancement of autonomy in clinical decision-making. The proposed applications of AI “would result in more accurate predictions than existing methods” [26] and, thereby, “increase the chances that decisionally incapacitated patients receive the treatments they want and avoid the treatments they do not want” [27]. This is held as especially important in the numerous cases lacking an available and relevant advance directive, since the alternative strategy of surrogate-supported decision-making “often fails to provide treatment consistent with the patient’s preferences” [28]. Artificial Intelligence tools are also seen as having “potential to improve the transparency of ethical decision-making” [29] and, thereby, improving surrogate decision-making [28, 30], enabling new lines of action for clinicians [31], and boosting respect for the autonomy of all stakeholders in the process [32, 33]. On the flipside, some ethicists fear the opposite: by reducing the ethical deliberation process to the statistical correlation found in the training data that underlie the ML tools [27, 34], and, thus, deploying social and demographic features as sole determinants of their preferences, AI “could potentially endanger patient autonomy” [35]. Artificial Intelligence could lead to conflicted intuition in stakeholders by giving supportive information based on wrong assumptions [28, 36, 37], or not being more accurate than the surrogates predictions [29, 38] (which critics think is likely because the “unpredictable instability of preferences inherently limits any prediction model” [38]).

It is also assumed that AI could improve the benefits one can receive through clinical ethical decision-making by supplying reliable information, such as “offering evidence of the patient’s preferences” [39], to support clinicians, surrogates or other stakeholders. This would be beneficial to the general quality of the clinical treatment and the decision-making process as a whole [24, 28]. The additional reassurance may “help to relieve some of the burdens associated with making decisions for incapacitated patients” [23] especially for the surrogates (but also for healthcare personnel and clinical ethicists). These burdening situations do not only arise in large clinics where CESS is readily available, but also in small hospitals or primary care. Having ethical AI as an easily accessible tool on every digital terminal device enables this kind of ethical support for a broader range of users and situations [26, 40], with the potential of saving the healthcare system human and economic resources [26, 41]. Furthermore, the use of AI in the clinical setting may provide a form of cognitive moral enhancement [42] and promote ethical competencies [43]. On the other hand, critics argue that the information supplied by AI may not be as robust as one might think, since “even well-performing algorithms can be unreliable in individual cases” [24]. The algorithms on which the tools are based may never be fully comprehensive of the actual ethical decision-making process, as the complex deliberations are “unlikely to be successfully reduced to a set of equations” [43]. Furthermore, AI is thought to lack the ability to act empathetically [40] or take structural and systematic knowledge into account, as “context and explanations are still hard for algorithms to grasp” [24]. If that holds true, the use of AI may pose no clear benefit [41], while still potentially undermining the competencies of the stakeholders [44].

Artificial Intelligence may be helpful in terms of non-maleficence by adding to the conventional methods of ethical decision-making, which otherwise “places significant stress on surrogate decision makers”[28]. The support could occur potentially in the form of advice that informs and supports the stakeholders who “may be ill-prepared for the high stakes decisions they find themselves needing to make”[39], and “often have difficulty distinguishing their preferences from the patient’s preferences” [45]. On the contrary, some authors fear that the applications in question “might increase the stress on some surrogate decision makers” [32] by undermining their confidence in cases in which the AI does not agree with their assessment of the situation [39], or by re-enforcing cognitive biases and flaws in their decision-making [46]. Another “unfortunate consequence is that it will likely limit the development of the ethical sensitivity otherwise obtained through engaging with challenging ethical cases”[31], which can lead to de-skilling.

Some authors have hopes for AI to increase the justice and fairness of clinical decision-making by decreasing biases [25] and “provid[ing] an objective, accurate, and individualized assessment” of challenging cases [25]. Having said that, the majority of reasons extracted point in a different direction: as a direct consequence of the choice of respective training data, the use of AI could “simply reflect existing biases” [26] and, thus, “perpetuate social injustices” [47]. Additionally, healthcare institutions (hospitals, health insurance companies, industry) may implement new biases “to make predictions that are favorable for the hospital budget” [32].

Explicability was of less concern in the ethical debate than the other principles. The reasons mentioned focused on the lack of transparency [35], explicability [35] and accountability in AI-supported decisions [31]. While “concerns about trusting a “black box” have been expressed” [24], most of the publications reviewed did not mention any related factors.

Some reasons that did not fit any of the biomedical principles did occur, most of them focused directly on shortcomings in the development of the advisory applications. Issues of the AI being developed based on wrong assumptions or insufficient amounts of data [37, 47], the development being too resource-intensive [48] or that “the automation of ethical decision-making using AI is currently neither feasible nor ethical” [49] were mentioned as causes to dismiss its implementation. In addition, decision-making by surrogates was in itself described as superior to AI alternatives [38].

## Discussion

The CDSS are already well-established in clinical practice and have been widely discussed in the scientific community. It is, therefore, natural to compare the results of this review with the discussion on clinical support systems as the gold standard.

The results show an uneven distribution of references to different ethical principles, with positive and negative aspects of autonomy being by far the most frequently mentioned. The high occurrence of these arguments is due probably to the fact that most of the AI tools included in the review work directly with predictions of patient preferences. These applications are deeply intertwined with issues of patient and stakeholder autonomy, leading to a wide variety of arguments about autonomy.

The low occurrence of references to justice came as a surprise, as the topic is frequently discussed in the ethical debate on “traditional” CDSS which are not specifically directed at *ethical* decision-making [50]. One reason for the lack of references to justice might be linked to the fact that the developers of METHAD (as one of the main applications examined in this review) decided not to encompass the principle of justice into their algorithm. Another reason could be the contextual dependence of the concept of justice, especially in the various healthcare systems worldwide. Justice may be interpreted completely different in systems in which healthcare is seen as a right, where the equitable distribution of resources is one of the main concerns. In systems assessing healthcare as a commodity, by contrast, the access to the resources itself serves as a bottleneck, and the issue of justice loses importance on the individual level. The surplus of publications included from countries with “commodity-based” healthcare systems potentially underlies the lack of references to justice.

Other aspects of utmost importance in the discussion on CDSS had a surprisingly small impact on the debate regarding their ethical counterpart. Although biases were mentioned as an issue, they were generally of less concern in the ethical support systems than they are typically in diagnostic decision support [51]. The topic of man–machine-interaction was virtually non-existent in the ethical debate, whereas it is an essential part of the discussion on CDSS [52, 53].

The explicability of AI-supported decision-making as a whole was seemingly not of concern to most of the scientific community. In the general ethical debate on AI, explicability and related values, such as transparency and explainability, are indeed the most common principles even before the traditional ones of biomedical ethics [54]. Explainability seems particularly crucial in situations when the application of black-box AI systems justifies diagnostic or therapeutic procedures with severe implications or because stakeholders may desire to understand how a decision has been derived [21]. The possibility of explicability being thought of as irrelevant, in either positive or negative terms, may be possible, though highly unlikely and possibly disadvantageous to further research.

Many of the reasons given are not new; for example, issues of de-skilling, transparency of recommendations and bias in the development of numerous tools are frequently discussed in debates about the use of conventional algorithms to support decision-making or the digitalisation of healthcare in general. Reasons that only arise in the context of AI, such as the availability of sufficient training data to develop reliable algorithms, or questions about trusting a “black box” trained by statistical learning methods, were far outnumbered.

We assume that “human” clinical ethics support is the gold standard for support systems in clinical ethical settings. The tools discussed in this review are being developed to support, or even undertake, tasks that have, so far, been part of the work of ethics support structures. They attempt to improve the quality and availability of support concerning ethically challenging cases for patients, physicians and other stakeholders; but there is an epistemic hurdle to measure their benefits. A comparative evaluation of human and machine-derived ethics support is difficult, as there is an ongoing debate on appropriate outcome parameters for measuring ethical quality even in “conventional” CESS [55]. As long as there is no way to quantify the value of the existing systems, it is hard to determine the impact of new ones.

We acknowledge the limitations of the current work. As this review has been carried out at a relatively early stage in the life cycle of the subject, it should be seen as a baseline inquiry. With time and scientific progress, more and more AI tools will emerge, along with more ethical arguments. The focus on only five conjectured applications, of which only two provide the bulk of the reasons given, is one of the main shortcomings of this review. Most of these algorithms focus on patient autonomy, which has the potential to bias the results of this review. In addition, only one of the tools has been concretely developed and only used in hypothetical settings. Any additional issues relating to development, technical feasibility, application and human–machine interaction can only be speculated about at this stage. These shortcomings may only be remedied with time, further development and the use of the tools in question. At a later date, this review should be repeated; the quantity and kinetics of the results compared with the present ones should allow for interesting conclusions to be drawn.

Furthermore, it is important to acknowledge that in a more and more interconnected and globalised world, especially when exploring topics with important ethical and technological components, one should pay attention to different cultural contexts, their inherent differences in ethical tendencies as well as diverging levels of acceptance of new technologies. As the publications included depict the US-American and European perspectives almost exclusively this could be seen as a limitation of the validity of our findings. Furthermore, the reasons identified in our review are partly embedded in the frameworks of different ethical theories which has an impact on their correct understanding. Starting from principlism as an analytic framework, however, we see a chance for integrating different ethico-theoretical accounts in one set of arguments.

## Conclusions

The prospective benefits of the use of AI in clinical ethical decision-making are manifold. The use of such tools can have a positive impact on individual patients, surrogates, physicians, healthcare staff and the healthcare system as a whole. However, a number of drawbacks need to be addressed before such systems can be implemented and used in a clinical context. While issues concerning all of the principles of medical ethics were brought up, the publications reviewed were largely focused on autonomy. We believe that justice, as a fundamental and universal value, has far-reaching implications for the development and use of AI, and, therefore, can and should play a greater role in the future development of AI-driven ethical support systems. The additional AI-related principle of explicability was not sufficiently discussed either, and should find its way into scientific deliberations more frequently.

Other issues that are more prevalent in the debate on CESS, such as bias and human–machine interaction, were rarely explored in the publications reviewed. While further progress in the development of ethical AI tools is needed to explore practical consequences, proactively discussing these issues and, thus, guiding design along ethical pathways should be a priority for the scientific community.

## Supplementary Information

**Additional file 1. **Included publications.

**Additional file 2. **List of codes.

**Additional file 3. **ThePRISMA-Ethics Reporting Guideline.

## Data Availability

The datasets used and analysed during the current study are available from the corresponding author on reasonable request.

## Abbreviations

AI: Artificial Intelligence
ML: Machine Learning
CESS: Clinical Ethical Support Services
CDSS: Clinical Decision Support Systems
