# Supplementary material for: Should Artificial Intelligence be used to support clinical ethical decision-making? A systematic review of reasons
Source: BMC Med Ethics. 2023 Jul 6;24:48. doi: 10.1186/s12910-023-00929-6 (PMC10327319; doi:10.1186/s12910-023-00929-6)
Supplement: Supplementary file 1 — Additional file 1. Included publications. [file 12910_2023_929_MOESM1_ESM.pdf]

Additional File: Included Publications

1 Biller-Andorno N, Ferrario A, Gloeckler S. In search of a mission: Artificial intelligence in clinical ethics. Am J Bioeth 2022;22:23-5 doi:10.1080/15265161.2022.2075055.

2 Binkley CE, Kemp DS, Braud Scully B. Should We Rely on AI to Help Avoid Bias in Patient Selection for Major Surgery?. AMA J Ethics 2022;24:773 doi:amajethics.2022.773.

3 Ferrario A, Gloeckler S, Biller-Andorno N. Ethics of the algorithmic prediction of goal of care preferences: from theory to practice. J Med Ethics 2022;0 doi:10.1136/jme-2022-108371.

4 Howard D, Rivlin A, Candilis P, et al. Surrogate Perspectives on Patient Preference Predictors: Good Idea, but I Should Decide How They Are Used. AJOB Empirical Bioethics 2022;13:125 doi:10.1080/23294515.2022.2040643.

5 Wendler D. Promoting the Values for Surrogate Decision-making. JAMA : the journal of the American Medical Association 2022;328:243-4 doi:10.1001/jama.2022.10347.

6 Rid A. Will a patient preference predictor improve treatment decision making for incapacitated patients?. J Med Philos 2014;39:99-103 doi:10.1093/jmp/jhu001.

7 Ditto PH, Clark CJ. Predicting end-of-life treatment preferences: perils and practicalities. J Med Philos 2014;39:196-204 doi:10.1093/jmp/jhu007.

8 Sharadin NP. Patient preference predictors and the problem of naked statistical evidence. J Med Ethics 2018;44:857-62 doi:10.1136/medethics-2017-104509.

9 Shalowitz DI, Garrett-Mayer E, Wendler D. How should treatment decisions be made for incapacitated patients, and why?. PLoS Med 2007;4:e35 doi:10.1371/journal.pmed.0040035.

10 John S. Patient Preference Predictors, Apt Categorization, and Respect for Autonomy. J Med Philos 2014;39:169-77 doi:10.1093/jmp/jhu008.

11 Brock DW. Reflections on the patient preference predictor proposal. J Med Philos 2014;39:153-60 doi:10.1093/jmp/jhu002.

12 Lindemann H, Nelson JL. The surrogate's authority. J Med Philos 2014;39:161-8 doi:10.1093/jmp/jhu003.

13 Dresser R. Law, ethics, and the patient preference predictor. J Med Philos 2014;39:178-86 doi:10.1093/jmp/jhu004.

14 Kim SY. Improving medical decisions for incapacitated persons: does focusing on "accurate predictions" lead to an inaccurate picture?. J Med Philos 2014;39:187-95 doi:10.1093/jmp/jhu010.

15 Wendler D, Wesley B, Pavlick M, et al. A new method for making treatment decisions for incapacitated patients: what do patients think about the use of a patient preference predictor?. J Med Ethics 2016;42:235-41 doi:10.1136/medethics-2015-103001.

16 Rid A, Wendler D. Treatment Decision Making for Incapacitated Patients: Is Development and Use of a Patient Preference Predictor Feasible?. J Med Philos 2014;39:130-52 doi:10.1093/jmp/jhu006.

17 Rid A, Wendler D. Use of a patient preference predictor to help make medical decisions for incapacitated patients. J Med Philos 2014;39:104-29 doi:10.1093/jmp/jhu001.

18 Meier LJ, Hein A, Diepold K, et al. Clinical Ethics - To Compute, or Not to Compute?. Am J Bioeth 2022:1-4 doi:10.1080/15265161.2022.2127970.

19 Gundersen T, Bærøe K. Ethical Algorithmic Advice: Some Reasons to Pause and Think Twice. American journal of bioethics 2022;22:26-8 doi:10.1080/15265161.2022.2075053.

20 Kasperbauer TJ. Conflicting roles for humans in learning health systems and AI-enabled healthcare. J Eval Clin Pract 2021;27:537-42 doi:10.1111/jep.13510.

21 Earp BD. Meta-surrogate decision making and artificial intelligence. J. Med. Ethics 2022;48:287-9 doi:10.1136/medethics-2022-108307.

22 Um S. Autonomy, shared agency and prediction. J Med Ethics 2022;48:313-4 doi:10.1136/medethics-2022-108289.

23 Pilkington B, Binkley C. Disproof of Concept: Resolving Ethical Dilemmas Using Algorithms. Am J Bioeth 2022;22 doi:10.1080/15265161.2022.2087789.

24 Coin A, Dubljević V. Using Algorithms to Make Ethical Judgements: METHAD vs. the ADC Model. Am J Bioeth 2022;22:41-3 doi:10.1080/15265161.2022.2075967.

25 Char D. Important Design Questions for Algorithmic Ethics Consultation. Am J Bioeth 2022;22:38-40 doi:10.1080/15265161.2022.2075054.

26 Chambers T. An All-Too-Human Enterprise. Am J Bioeth 2022;22 doi:10.1080/15265161.2022.2075969.

27 Rahimzadeh V, Lawson J, Baek J, et al. Automating Justice: An Ethical Responsibility of Computational Bioethics. Am J Bioeth 2022;22:30-3 doi:10.1080/15265161.2022.2075051.

28 Sauerbrei A, Hallowell N, Kerasidou A. AIgorithmic Ethics: A Technically Sweet Solution to a Non-Problem. Am J Bioeth 2022;22:28-30 doi:10.1080/15265161.2022.2075050.

29 DeMarco JP, Ford PJ, Rose SL. Implicit Fuzzy Specifications, Inferior to Explicit Balancing. Am J Bioeth 2022;22:21-3 doi:10.1080/15265161.2022.2075970.

30 O'Neil CC. Commentary on 'Autonomy-based criticisms of the patient preference predictor'. J Med Ethics 2022;48:medethics-316.

31 Schwan BB. Sovereignty, authenticity and the patient preference predictor Comment. J Med Ethics 2022;48:311-2 doi:10.1136/medethics-2022-108292.

32 Mainz JT. The Patient preference predictor and the objection from higher-order preferences. J Med Ethics 2022:medethics-2022-108427 doi:10.1136/jme-2022-108427.

33 Crossnohere NL, Childerhose JE, Bose-Brill S. Increasing the Patient-Centeredness of Predictive Analytics Tools. PATIENT 2022;15:615 doi:10.1007/s40271-022-00595-7.

34 Sabatello M. Wrongful Birth: AI-Tools for Moral Decisions in Clinical Care in the Absence of Disability Ethics. Am J Bioeth 2022;22:43-6 doi:10.1080/15265161.2022.2075971.

35 Biller-Andorno N, Ferrario A, Joebges S, et al. AI support for ethical decision-making around resuscitation: proceed with care. J Med Ethics 2022;48:175 doi:10.1136/medethics-2020-106786.

36 Klugman CM, Gerke S. Rise of the Bioethics AI: Curse or Blessing?. Am J Bioeth 2022;22:35-7 doi:10.1080/15265161.2022.2075056.

37 Mast L. Against autonomy: How proposed solutions to the problems of living wills forgot its underlying principle. Bioethics 2020;34:264-71 doi:10.1111/bioe.12665.

38 Jardas EJ, Wasserman D, Wendler D. Autonomy-based criticisms of the patient preference predictor. J Med Ethics 2022;48:304-10 doi:10.1136/medethics-2021-107629.

39 Hubbard R, Greenblum J. Surrogates and Artificial Intelligence: Why AI Trumps Family. Sci Eng Ethics 2020;26:3217-27 doi:10.1007/s11948-020-00266-6.

40 Meier LJ, Hein A, Diepold K, et al. Algorithms for Ethical Decision-Making in the Clinic: A Proof of Concept. Am J Bioeth 2022;22:4 doi:10.1080/15265161.2022.2040647.

41 Barwise A, Pickering B. The AI Needed for Ethical Decision Making Does Not Exist. Am J Bioeth 2022;22:46-9 doi:10.1080/15265161.2022.2075052.

42 Biller-Andorno N, Biller A;. Algorithm-Aided Prediction of Patient Preferences — An Ethics Sneak Peek. N Engl J Med 2019;381:1480 doi:10.1056/nejmms1904869.

43 Demaree-Cotton J, Earp BD, Savulescu J. How to Use AI Ethically for Ethical Decision-Making. Am J Bioeth 2022;22:1-3 doi:10.1080/15265161.2022.2075968.

44 Lamanna C, Byrne L. Should Artificial Intelligence Augment Medical Decision Making? The Case for an Autonomy Algorithm. AMA J Ethics 2018;20:902 doi:amajethics.2018.902.
