# Supplementary material for: Should Artificial Intelligence be used to support clinical ethical decision-making? A systematic review of reasons
Source: BMC Med Ethics. 2023 Jul 6;24:48. doi: 10.1186/s12910-023-00929-6 (PMC10327319; doi:10.1186/s12910-023-00929-6)
Supplement: Supplementary file 2 — Additional file 2. List of codes. [file 12910_2023_929_MOESM2_ESM.pdf]

List of Codes

Topic				Codings	included in (numbers according to supplement "included publications")
				552	
Autonomy				273	
	Prediction Accuracy				
		pro			
			Superior accuracy of predictions compared to existing methods	48	2, 5, 6, 15, 16, 18, 19, 20, 21, 22, 23, 25, 28, 31, 34, 35, 36, 40, 43, 44
			Surrogates predict patient preferences incorrectly	20	4, 6, 16, 18, 19, 25, 34, 36, 41, 43, 44
			Surrogates predict preference no more accurate than chance	13	18, 19, 20, 23, 25, 26, 28, 31, 35, 36,
			AI helps to improve surrogate decision-making	8	4, 18, 20, 21, 36
			AI prediction is valuable information, even if not 100% accurate	3	2, 20, 21
			Surrogates' predicitons less accurate than AI predictions	1	21
			Patient Preference isn't detected through conventional methods	1	36
			Advance directive more likely to be outdated than AI-Predictions	1	36
		contra			
			AI does not improve accuracy (on controversial cases)	7	12, 20, 23
			Decisions have to be made for the right reasons	5	15, 21
			Previously expressed values do not equal later choices	5	14, 32, 34, 36
			Instability of preferences limits any prediction model	4	13, 23, 34
			Accuracy of predictions is not as important as well-being	3	6, 18
			Unlikely for AI-prediction to match ground-truth completely	3	2, 28
			Accuracy of Prediction models impossible to be validated	2	23
			AI can only be accurate with sufficient information on patient	2	18, 35
			Prediction susceptible to forecasting errors	1	28
			Accuracy of predictions is not as important as who decides	1	26
			AI accuracy cant be verified in incapacitated patients	1	36
			AI predictions may be broader than clinical case requires	1	35
	Autonomy of decision makers				
		pro			
			AI improves transparency of ethical decision-making	4	12, 29
			Systems augment deliberative capacity	4	5, 25
			AI can expand the available lines of action for clinicians	1	17
			AI helps stakeholders understand decision of similar people	1	4
		contra			
			AI might undermine the role of the ethicist	3	8, 9
			AI might undermine the role of surrogates	3	16, 35
			Stakeholders can manipulate AI to their own advantage	1	35
			AI will not be entirely controllable	1	29

List of Codes

Conventional surrogate decision-making poses negative implications for patients		
pro		
Use of AI can help avoid unwanted treatment	4	4, 20, 25
Analysis of Health Data		
pro		
AI has to be trained just once	1	25
Wider range of predictors than Human-run studies	1	25
Larger data samples than human-run studies	1	25
Faster analysis than human-run studies	1	25
contra		
Ethics consultation data is not plentyful enough to require AI	2	8
Respect for Autonomy		
pro		
High acceptance of AI in stakeholders	11	16, 18, 19, 20, 35, 44
AI can ensure that patients get the treatment they prefer	10	16, 18, 20, 34, 35, 41
Improved respect for Autonomy	8	13, 15, 20, 25, 35, 36
Empirical Prediction > "best interest" abstraction	2	15, 20
AI-advice can enhance patient autonomy	2	2, 36
AI predictions supply morally acceptable basis for decision-making	1	14
Informed patients would not want to overburden their family	1	36
Deferring to AI does not undermine deliberative capacity	1	19
Patient autonomy > having a family member decide	1	19
Sense of identity (provided by AI) > best interests prediction	1	15
AI recommendations can provide legitimation for actions	1	2
AI can help clinicians to protect patient autonomy	1	35
contra		
Process could limit patients' autonomy	12	4, 16, 20, 22, 28, 32, 36, 39
Statistical correlation does not equal personal decision	10	15, 16, 20, 23, 28, 41, 44
The right choice being made is not sufficient for autonomy	6	20, 23, 27, 32, 34
Some patients may not have preferences to predict	5	23, 34
Entrusting surrogates with decisions is a form of autonomy	4	23, 34
Automating the decision-making-process is dehumanizing	3	13, 19
Predicting someones preferences limits their autonomy	2	41, 42
AI can enforce stereotyping	2	21, 36
Low acceptance of ethics AI tools in general public	2	20, 29
Surrogates more likely decide based on reasons patients endorse	2	21, 34
Low acceptance of ethics AI tools in clinicians	1	2

List of Codes

Systematic use of AI may harm autonomy significantly	1	39
Making decisions yourself is an essential part of autonomy	1	26
Accuracy does not legitimize predictions	1	23
Surrogates		
pro		
Advance directives fail to match treatment to preferences	3	16, 35, 36
Advance directive are oftentimes not available	3	16, 34, 35
Reliance on family based on wrong assumption of accuracy	2	21, 36
"the autonomy algorithm is truly patient centered"	2	25
Physicians fail to match treatment plan to patients preference	2	25, 36
Surrogates may have limited knowledge of preferences	1	16
Advance directives may not include relevant information	1	16
Only a part of Patients want their families to decide for them	1	20
contra		
The Family should be in charge of decisions, not AI	4	19, 20, 40, 44
What is best for the loved ones has to be considered in decision-making	2	6, 20
Surrogates should be in charge of decisions, not AI	2	18
Beneficence	128	
Wellbeing of Surrogates		
pro		
Reduced emotional strain on Surrogates	19	4, 16, 18, 19, 20, 25, 26, 34, 35, 36, 40, 44
"offer relief from burden posed by incapacitated patients"	6	13, 25, 35, 36, 44
surrogate "rest a little easier" when AI supports the decision	3	25, 35, 36
AI helps surrogates not to feel alone with decisions	2	16, 18
AI facilitates cognitive burden for doctors	1	2
Reduced burnout of surrogates	1	25
"An algorithm will not lose sleep" (over important decisions)	1	25
contra		
AI does not decrease surrogates' decisional burden	1	20
Economic Considerations		
pro		
AI saves healthcare resources	5	8, 17, 28, 38
Existing methods are resource intensive	4	25, 28
Economic Gains for society	2	25
contra		
Ethicists' labor costs not significant enough to be reduced	1	8
Surrogates		

List of Codes

contra			
	Surrogates concerned that AI does not reflect the individual	1	35
	Overriding the familial surrogate right is impermissible	1	19
	Families as surrogates have great interest in patients' good	1	19
Normative Enhancement			
pro			
	"Cognitive moral enhancement"	5	11, 30, 33, 38
	AI may advance the study of ethical theory	4	2, 11, 30, 38
	Computational extension of moral methods	3	11, 22
	AI provides normative transparency	2	11, 30
	AI constitutes a way to train our "moral orientation"	1	2
	AI could promote rational dissensus	1	12
	AI can promote the common good	1	37
contra			
	Normative enhancement through AI is unnecessary	1	38
Quality of Support			
pro			
	AI might improve the process of surrogate decision-making	8	2, 14, 18, 35, 36, 44
	AI enhances availability of ethical support	4	12, 20, 24, 25
	AI predictions enable faster treatment decisions	4	16, 35, 36
	AI offers new factors to consider in surrogate decision-making	4	16, 18
	AI is faster than human intelligence	3	7, 20, 33
	Processing power enables better support	3	4, 37
	AI has better judgement than singular clinician/committee	3	12, 19
	AI may help in otherwise overwhelming situations	3	4, 17, 29
	Exisiting methods consider only small patient cohorts	2	25
	AI aims to facilitate informed decision-making	2	10, 36
	AI is more consistent than human intelligence	2	7, 33
	AI may increase physician and patient wellfare	1	36
	AI predicitions are easy and fast to document	1	2
	AI enables "wisdom of crowds" for individual decision-making	1	2
contra			
	AI decision-making is not fully comprehensive of moral processes	6	8, 29, 30, 38, 42, 43
	Assisstive AI poses no clear benefit	2	2, 8
	AI is unable to act empathetic	2	24, 30
	AI cant feel "moral regret and residual obigation"	2	7, 30
	Human input is essential for ethical tasks	1	3

List of Codes

	Humans are better suited for actual ethic work	1	3
	AI can be unreliable	1	2
	AI decision-making inferior to human decision-making	1	29
	AI is unable to take structural knowledge into account	1	2
	AI could learn to emulate ethics committees, but not exceed them	1	12
	AI does not spare time	1	38
	Reliance on AI can undermine the providers competency	1	37
Non-Maleficence		56	
Conventional methods pose health risks for stakeholders			
pro			
	Surrogate stress, mental health problems	19	4, 13, 16, 18, 20, 25, 26, 34, 35, 36, 43, 44
	Surrogate decision-making takes time, delays prefered treatment	1	36
	Conventional methods induce health care professional burnout	1	25
contra			
	AI may increase surrogate stress	6	13, 16, 26, 35
decision-making-processes			
contra			
	De-Skilling	3	2, 17, 30
	AI can enforce cognitive biases	2	6
	AI can reinforce current flaws in decision-making	2	4, 6
	AI tools can be used to deny expensive care	1	18
	Having choices by patients with capacity imposed on others could harm them	1	14
	AI does not care about others	1	7
	negative impact on complex human communication	1	38
	AI can introduce new flaws in decision-making	1	4
	AI may "streamline" ethical advancement	1	38
Surrogates			
pro			
	Surrogates mistake their own for the patients' preferences	2	16, 19
	Surrogates might be ill-prepared for their role	2	16, 18
	Surrogate decision-making biased by interaction with other loved ones	1	16
	AI keeps surrogates from bruteforcing their own preferences	1	18
	AI reduces distorting biases in Surrogates	1	36
	Surrogate decision-making leads to the patients perspective being excluded	1	43
	Surrogate may have psychological conditions impairing judgement	1	19
	Surrogates do not understand the patients clinical status	1	19
	Surrogates do not understand their role	1	18

List of Codes

	contra				
		AI may increase decision-making burden	2	18, 35
		AI may undermine surrogates' decisional confidence	2	26, 35
		Surrogate bias may be adpoted by AI	1	35
Justice			55	
Bias				
	pro			
		AI can decrease biases in decision-making	6	4, 5, 16, 19
		Conventional methods introduce bias	2	5, 35
	contra			
		Machine learning tools can simply reflect existing biases	13	2, 4, 5, 16, 18, 21, 24, 25, 28, 29, 44
		Automation bias	6	16, 24, 30, 33
		Institutions could intentionally implement biases	4	19, 35
		Disability Bias	2	37
		Algorithmic Bias	2	19, 24
		Anchoring bias	1	33
decision-making				
	pro			
		AI can increase fairness in decision-making	2	5, 33
		AI decision-making is objective	2	5
		AI-decisions not compromised by legal implications	1	4
		AI can mitigate racial inequalities	1	5
	contra			
		AI can enforce inequality	9	4, 10, 16, 18, 21, 25, 34
		AI decisions may require additional human input	3	16, 33, 38
		AI can decrease fairness in decision-making	1	16
Explicability			20	
decision-making				
	pro			
		AI decision-making doesn't have to be explicable as humans decision-making is neither	2	2, 30
	contra			
		Lack of transparency of AI	3	16, 17
		Lack of explicability of AI	3	16, 17
		AI does not improve transparency of ethical decision-making	2	29
		There are concerns about trusting a "black box"	2	2, 16
		Digital inputs into AI tools abstract the individual cases	1	35
Legal Considerations				

List of Codes

	contra				
		Lack of accountability of AI	3	4, 17, 30
		Responsibilty for information provided by AI is hard to assign	2	8
		There are no official certifications for AI	1	24
		Automation of ethical decision-making is currently not ethical	1	1
Other			20	
Processes				
	contra			
		Surrogate decision-making in itself is superior to AI alternatives	2	23
Development				
	pro			
		AI tools enable the collection of patient data	2	16
		Data collected by AI can be used for its development	1	16
	contra			
		AI is being trained based on wrong assumptions	3	14, 32
		Available data not sufficient to train AI to be superior	3	1, 13, 34
		Development is resource intensive	2	13, 28
		Automation of ethical decision-making is technically not feasible	2	1
		Subpar implementation of tools hinders motivation to use them	1	16
		Development costs outweigh benefits	1	34
		Successful algorithm may hinder its own development	1	17
		ML tools not yet fully realized	1	28
		The role of ethics consultant is difficult to capture in code	1	32
