# Supplementary material for: Should Artificial Intelligence be used to support clinical ethical decision-making? A systematic review of reasons
Source: BMC Med Ethics. 2023 Jul 6;24:48. doi: 10.1186/s12910-023-00929-6 (PMC10327319; doi:10.1186/s12910-023-00929-6)
Supplement: Supplementary file 3 — Additional file 3. ThePRISMA-Ethics Reporting Guideline. [file 12910_2023_929_MOESM3_ESM.pdf]

THE PRISMA-ETHICS REPORTING GUIDELINE		
**Title**		
1	**Title**: Identify the report as a systematic review.	✓
**Abstract**		
2	**Structured summary**: Provide a structured summary including, as applicable: objectives; ethics literature eligibility criteria; information sources; ethics literature appraisal and synthesis methods; included publications; synthesis of results; limitations of evidence; interpretation (conclusions and implications of key findings); funding; systematic review registration number.	✓ (registration number in declarations)
**Introduction**		
3	**Rationale**: Describe the rationale for the review in the context of existing knowledge.	✓
4	**Objectives:** Provide an explicit statement of the objective(s) or question(s) the review addresses.	✓
**Methods**		
5	**Eligibility criteria**: Specify the inclusion and exclusion criteria for the review (e.g., years considered, language, type of publication) and give a rationale.	✓
6	**Search strategy:** Specify all databases, registers, websites, organisations, reference lists and other sources searched or consulted to identify publications. Specify the date when each source was last searched or consulted. Provide the rationale for using the information sources and present the full search strategy, including any limits and filters used, such that it could be repeated.	✓
7	**Selection process**: Specify the methods used to decide whether a publication met the inclusion criteria of the review, including how many reviewers screened each record and each publication retrieved, whether they worked independently and how disagreements were resolved, and if applicable, details of automation tools used in the process.	✓
8	**Data extraction**: Indicate which sections of the publication were analysed and how were the data extracted from the publication. If applicable, state the software and details of automation tools used in the process.	✓
9	**Identification of codes and themes:** Explain the process of assigning the codes, themes, or items (e.g. inductive, deductive, a combination of deductive and inductive strategies), if applicable. If so, describe the process for coding of data (e.g. line by line coding to search for concepts), including how many reviewers analysed each publication. List and define all other variables for which information were sought (e.g. participant and intervention characteristics, funding sources). Describe any assumptions made about any missing or unclear information.	✓
10	**Quality appraisal:** Indicate whether a quality appraisal was performed and why, and if yes, outline the quality appraisal process and its results (e.g. how many reviewers assessed each study, did they work independently).	✓
11	**Synthesis methodology:** Identify the synthesis methodology or theoretical framework which underpins the synthesis, and describe the rationale for choice of methodology (e.g. thematic analysis, content analysis, critical interpretive synthesis, grounded theory synthesis, narrative synthesis). Describe any methods used to tabulate or visually display results of individual studies and syntheses.	✓

**Results**		
12	**Publication selection process**: Describe the results of the search and selection process, from the number of publications identified in the search to the number of studies included in the review, with a flow diagram (including reasons for exclusions at each stage).	✓
13	**Characteristics of publications**: For each publication, included in the review, present characteristics for which data were extracted and provide the citations.	For many, but not all publications
14	**Results of syntheses:** Present the results (e.g. new systematization of issues or arguments) and reference publications as evidence.	✓
15	**Quotations**: Provide original wording to illustrate themes, if applicable.	✓
**Discussion**		
16	**Summary**: Summarize the main findings and provide a general interpretation of the results in the context of other evidence; consider their relevance to key groups (e.g. health care workers, academics, other decision maker).	✓
17	**Strength and limitations**: Discuss strengths and limitations of the publications included in the review and the review process itself.	✓
18	**Conclusions**: Discuss implications of the results for practice, policy and/or future research.	✓
**Other Information**		
19	**Registration and protoco**l: State whether the review was registered, or state that the review was not registered. If yes, provide registration information for the review, including register name and registration number. Indicate where the review protocol can be accessed, or state that a protocol was not prepared. Describe and explain any amendments to information provided at registration or in the protocol.	✓
20	**Support:** Describe sources of funding for the systematic review and other support (e.g., supply of data); role of funders for the systematic review.	✓
21	**Competing interests:** Declare any competing interests of review authors.	✓
22	**Availability of data, code and other materials:** Report which of the following are publicly available and where they can be found: template information collection forms; information extracted from included studies; information used for all analyses; analytic code; any other materials used in the review.	✓
